# The role of exosomal lncRNAs in mediating apoptosis and inflammation in UV-induced skin photoaging

**DOI:** 10.3389/fcell.2025.1538197

**Published:** 2025-04-14

**Authors:** Kunjie Li, Songfa Lin, Pengjun Zhou, Yanni Guo, Shu Lin, Chao Ji

**Affiliations:** ^1^ Department of Dermatology, The Second Affiliated Hospital of Fujian Medical University, Quanzhou, Fujian, China; ^2^ Centre of Neurological and Metabolic Research, The Second Affiliated Hospital of Fujian Medical University, Quanzhou, Fujian, China; ^3^ Group of Neuroendocrinology, Garvan Institute of Medical Research, Darlinghurst, NSW, Australia; ^4^ Department of Dermatology, The First Affiliated Hospital of Fujian Medical University, Fuzhou, Fujian, China

**Keywords:** skin photoaging, exosome, lncRNA, rat, cis-regulatory

## Abstract

The skin, as the body’s largest organ, functions as a vital barrier against environmental insults. Chronic exposure to ultraviolet (UV) radiation significantly contributes to premature aging, or photoaging, which leads to DNA damage and disrupts repair mechanisms. Exosomes, which are small extracellular vesicles, play a key role in cell-to-cell communication and might help mitigate the effects of photoaging by transporting bioactive molecules to skin cells. Long non-coding RNAs (lncRNAs) are increasingly recognized for their regulatory roles in the photoaging process, influencing stress responses and DNA repair; however, their involvement in exosomes in the context of skin aging is not yet well understood. In this study, we developed a photoaging model using SD rats subjected to UVA and UVB irradiation, which led to significant changes in the dermis such as increased dryness, wrinkles, pigmentation, and vascular alterations. Histological evaluations showed uneven thickening of the epidermis, degradation of collagen and elastic fibers, and cellular infiltration. Exosomes isolated from the dermal tissues exposed to UV radiation displayed altered size distributions. Transcriptomic analyses of the UV-treated rats identified 2,332 lncRNAs and 5,906 mRNAs that were differentially expressed, revealing significant involvement in pathways related to oxidative stress, apoptosis, and cellular stress responses. A cis-regulatory analysis identified 1,327 essential interactions between lncRNAs and mRNAs, highlighting their role in controlling inflammation and apoptosis. Importantly, both *IL-1B* and *GADD45B* levels were significantly increased in the exosomes and UV-challenged HaCaT cells, indicating their crucial roles in responding to UV-induced stress. This study highlights the significant role of exosomal lncRNAs in managing cellular reactions to UV-induced stress, impacting regulatory pathways associated with apoptosis, inflammation, and oxidative stress. These insights pave the way for the development of lncRNA-focused therapeutic approaches to address UV-induced skin damage.

## Introduction

The skin, being the human body’s largest organ, serves as a critical barrier protecting against dehydration, radiation, mechanical damage, and infections (Parrado et al., 2019). It plays a critical role in safeguarding the body from deleterious environmental factors and exogenous substances. Skin aging is characterized by morphological changes that are influenced by both intrinsic and extrinsic factors. Intrinsic aging, also known as chronological aging, is a natural process that accompanies a gradual decline in physiological functions over time. In contrast, extrinsic aging is driven by environmental factors such as exposure to ultraviolet (UV) radiation, chemical pollutants, nutritional deficiencies, and lifestyle choices (Krutmann et al., 2021). Among these factors, prolonged UV exposure is a predominant contributor to premature skin aging, commonly referred to as photoaging.

Skin photoaging refers to the premature aging of the skin induced by ultraviolet (UV) exposure, characterized by reduced skin elasticity, increased wrinkle formation, and hyperpigmentation. UV exposure accelerates skin aging by triggering DNA damage, oxidative stress, and inflammatory responses. Ultraviolet A (UVA) radiation deeply penetrates the dermal layer, leading to indirect DNA damage and the breakdown of collagen and elastin fibers via oxidative stress pathways, thus significantly contributing to extrinsic aging (Sun et al., 2020). In contrast, ultraviolet B (UVB) radiation predominantly impacts the epidermis, inducing direct DNA damage via the formation of cyclobutane pyrimidine dimers (CPDs) and (6-4) photoproducts (6-4PPs), consequently activating apoptotic pathways and inflammatory responses (Steurer et al., 2019; Kajitani et al., 2022). Prolonged exposure to both UVA and UVB radiation increases NADPH oxidase activity, which boosts the production of reactive oxygen species (ROS). This increase in ROS not only intensifies inflammation but also triggers the release of cytokines and chemokines, further accelerating the aging of the skin (Fuller, 2019). Additionally, sustained UVB exposure exacerbates DNA damage and activates pathways such as p16(INK4a)/Rb and p53/p21(WAF1). These pathways promote the expression of the cyclin-dependent kinase inhibitor p16INK4a, leading to cell cycle arrest and impairing DNA repair mechanisms (Rastogi et al., 2010; Greussing et al., 2013; McAdam et al., 2016).

In the context of UV damage, exosomes play a vital role as key facilitators of intercellular communication, crucial in enhancing the skin’s protective mechanisms. These extracellular vesicles, typically around 100 nm in diameter, originate from the endosomal compartments of various cell types (Cheng and Hill, 2022). They are ubiquitously present in cell culture media and a plethora of biological fluids, including serum, plasma, urine, saliva, ascitic fluid, cerebrospinal fluid, amniotic fluid, pleural fluid, peritoneal fluid, and pericardial fluid (Safdar et al., 2016; Beltrami et al., 2017; Nazri et al., 2020; Wu et al., 2023). These exosomes are packed with a diverse array of biomolecules like DNA, RNA, non-coding RNA, lipids, and proteins, exosomes which enable them to facilitate complex intercellular communication, modulate immune responses, and inhibit apoptotic pathways (Gezer et al., 2014; Zhang et al., 2015; Tang et al., 2018; Abel et al., 2020; Monguió-Tortajada et al., 2022). In the context of skin aging, exosomes play a vital role in delivering bioactive molecules to dermal cells, significantly slowing aging effects and alleviating photoaging symptoms. They are known to promote collagen production in photoaged skin and help diminish hyperpigmentation (Thakur et al., 2023). When used in topical products such as creams, serums, oils, and masks, exosomes act as a protective layer and boost skin resilience (Yang et al., 2021). Thus, exosomes present a promising approach to preserving skin function and counteracting photoaging.

Long non-coding RNAs (lncRNAs) are known for their tissue- and cell-specific expression and play critical roles in various biological functions including cell proliferation, morphogenesis, pluripotency, development, neuronal activities, and gametogenesis (Guttman et al., 2009; Flicek et al., 2014). LncRNAs play pivotal roles in UV-induced DNA damage repair, oxidative stress, and inflammatory responses. For instance, certain lncRNAs regulate gene expression to modulate the response of skin cells to UV stress, such as by enhancing DNA repair mechanisms or suppressing the overexpression of pro-inflammatory cytokines (Zhang et al., 2019). Moreover, lncRNAs can mitigate cellular damage from ROS by regulating oxidative stress-related pathways (Liang et al., 2024). A growing body of evidence underscores the pivotal roles of lncRNAs in the modulation of photoaging. UV radiation can alter lncRNA expression, which then participates in cellular stress responses, activates p53, and supports DNA repair processes in skin cells. In dermal fibroblasts exposed to chronic UVA, several lncRNAs are differentially expressed, affecting collagen metabolism, epidermal differentiation, extracellular matrix (ECM) degradation, and crucial signaling pathways like MAPK and TGF-β (Piipponen et al., 2020). Additionally, lncRNAs play a role in UV-induced melanogenesis; for example, lnc-CD1D-2 is notably upregulated in UVB-irradiated melanocytes, where its increase, prompted by ROS, promotes melanin synthesis (Zeng et al., 2016).

Although the roles of lncRNAs in photoaging are increasingly recognized, their specific functions and regulatory mechanisms remain insufficiently understood. In particular, the roles of many UV exposure-related lncRNAs have yet to be fully characterized, especially their dynamic changes within exosomes and their contributions to photoaging processes. The objective of this study was to establish a rat model of skin photoaging, isolate skin-derived exosomes, and perform thorough sequencing of lncRNAs and mRNAs. Thereafter, through the analysis of exosomal lncRNA and mRNA alterations, we aimed to decipher the crucial roles of lncRNAs in orchestrating skin aging processes. These findings can enhance our comprehension of exosomal transformations during skin photoaging and potentially leading to novel exosome-based therapeutic approaches.

## Materials and methods

### Establishment of a rat model for UV-induced skin photoaging

Twenty male SD rats, aged approximately 14 weeks and weighing between 200 ± 20 g, were obtained from the Experimental Animal Center of Quanzhou Medical College. These rats were kept under controlled environmental conditions with a 12-h light/dark cycle and had access to standard lab food and water *ad libitum*. For histological studies, an Eclipse 80I optical microscope (Nikon Corporation) was used. A custom-built sunlight simulator was utilized for UV exposure, equipped with seven UVA lamps (Philips, 60W, spectral range 360–420 nm) and two UVB lamps (Philips, 40W, spectral range 280–320 nm). The rats were split into two groups: a UV group (n = 10) and a control group (n = 10). Before starting the irradiation, the dorsal fur of each rat in the UV group was shaved across a 5.0 cm × 10.0 cm area using an electric pet clipper. The cages were placed 30 cm away from the lamps to ensure even exposure. Both UVA and UVB lamps were activated simultaneously to mimic natural sunlight, with a total exposure time of 90 min per day. Over the 120-day exposure period, daily observations of the rats’ dorsal skin were made for signs of photoaging, such as thickening and wrinkling. The total UVA irradiation amounted to 200 J/cm^2^ and UVB exposure reached 30 J/cm^2^. Post-exposure, UV irradiation was discontinued, and the skin underwent histopathological analysis to verify the successful creation of the photoaging model.

### Extraction and characterization of exosomes from rat dorsal skin tissues

Dorsal skin samples from rats were enzymatically dissociated overnight at 4°C using 2.4 U/mL Dispase II and subsequently washed with phosphate-buffered saline (PBS). The samples were then finely chopped into 1 mm^3^ pieces and digested with 0.2% Type I collagenase at 37°C for 2–3 h. To stop the digestion, DMEM with 10% exosome-depleted serum was added. The resulting mixture underwent a series of centrifugations: initially at 2000 rpm for 5 min, followed by 2000 × g for 30 min, and then at 10,000 × g for 45 min to eliminate larger vesicles. Subsequently, the supernatant was passed through a 0.45 μm filter and ultracentrifuged at 100,000 × g for 70 min at 4°C. The collected pellet was resuspended in chilled PBS and ultracentrifuged again at the same settings. The final pellet was prepared for analysis using transmission electron microscopy (TEM) (JEOL Ltd., Tokyo, Japan), nanoparticle tracking analysis (NTA) (ZetaView, Particle Matrix, Meerbusch, Germany), and nanoflow cytometry for examining exosomal surface protein expression.

### RNA extraction from exosomes and quality control

RNA lysis buffer was added to the thawed exosome extract, followed by 140 µL of chloroform. The mixture was incubated at room temperature for 3 min and then centrifuged at 4°C, 12,000 g for 15 min. The supernatant was carefully decanted into a RNeasy spin column. The column was washed first with Buffer RWT and then with Buffer RPE. RNA was subsequently eluted using RNase-free water and the extracted RNA was immediately frozen at −80°C. Any exosomes not used were also stored at −80°C. The purity of the isolated RNA was assessed by its absorbance ratio at 260 nm–280 nm, and its quality was evaluated using a Nanodrop ND-1000 system (Thermo Fisher Scientific, CA). The RNA integrity was verified using an Agilent Bioanalyzer 2,100 (Agilent Technologies, United States).

### Library construction and sequencing

Library construction and sequencing were performed according to previously described methods (Williams et al., 2013; Iyer et al., 2015). The transcriptome libraries were assembled according to the manufacturer’s instructions using the Lifeint Transpose DNA Library Prep Kit and Lifeint Library Prep Index Kit for Illumina. Sequencing was carried out on an Illumina platform, generating 150 nucleotide paired-end reads.

### Transcriptome data processing

Quality control measures were applied using Fastp to eliminate low-quality data, which included trimming adapter sequences, primers, ambiguous bases (Ns), and reads with quality scores under 20. Reads that were less than 20 bp post-trimming were discarded. The high-quality reads were then aligned to the *Rattus norvegicus* genome (mRatBN7.2) using STAR aligner (Dobin et al., 2013). Transcript assembly was performed with Stringtie (Pertea et al., 2016), where alignment files helped to build a comprehensive transcriptome. This transcriptome was compared to human genome annotations with gffcompare to identify both annotated and potential lncRNA transcripts exceeding 200 nt. The coding potential of these lncRNAs was evaluated using CPC2, CNCI, Pfam, and FEElnc, retaining those predicted as non-coding by at least three of these tools. Both known and novel lncRNAs were quantified and annotated using featureCounts.Differential expression analysis was performed using DESeq2 (Love et al., 2014), with significance thresholds set at *FDR* < 0.05 and |logFC| ≥ 1. Default settings were maintained across all software applications.

### Analysis of gene ontology (GO) and kyoto encyclopedia of genes and genomes (KEGG) pathways

The enrichment analysis of Gene Ontology (GO) terms and KEGG pathways was carried out using the clusterProfiler package (Wu et al., 2021) (version 4.2) with a q-value cutoff of 0.05.

### Predicting *cis* action of hypothetical lncRNAs

To explore potential cis interactions involving hypothetical lncRNAs, we initially performed a correlation analysis based on the expression levels (TPM values) of differentially expressed lncRNAs and mRNAs. Significant interactions were determined when the Pearson correlation coefficient (|*r*|) was ≥0.80 and (*P* < 0.05). LncRNA-mRNA interactions were categorized as cis-acting if the target mRNA was found within a 100 kb window either upstream or downstream of the lncRNA site.

### Cell culture

HaCaT human immortalized keratinocytes were obtained and grown in Dulbecco’s Modified Eagle Medium (DMEM) enriched with 10% fetal bovine serum (FBS). The cells were kept at 37°C in a humidified environment with 5% CO_2_. To maintain cells in the logarithmic phase of growth, subculturing was carried out every 2–3 days.

### Transient transfection

Cells in the logarithmic phase of growth were adjusted to a density of 3 × 10^4^ cells/mL and plated into 6-well plates at 2 mL per well. When cells reached 70%–80% confluence, transfections were carried out using the following siRNAs: siR NC, siRNA-IL1B-1, siRNA-IL1B-2, siRNA-IL1B-3, siRNA-GADD45b-1, siRNA-GADD45b-2, siRNA-GADD45b-3, and overexpression constructs OE IL-1B, and OE GADD45b. Six hours after transfection, the medium was changed to fresh complete medium containing 10% FBS. Twenty-four hours post-transfection, the cells were harvested, washed twice with PBS, and then stored at −80°C for subsequent analysis.

### RNA isolation and RT-qPCR

Total RNA was isolated from the samples using TRIzol reagent (Invitrogen, United States) following the guidelines provided by the manufacturer. The sequences for the qPCR primers are listed in the Supplementary Table S5.

### Western blot analysis

Cells were lysed in RIPA buffer containing protease inhibitors. Protein concentrations were determined using a BCA protein assay kit. Equal amounts of protein were loaded for SDS-PAGE and then transferred to PVDF membranes. The membranes were incubated overnight at 4°C with primary antibodies: IL-1B Polyclonal antibody (26048-1-AP, 1:1,000, Proteintech, China), and GADD45B antibody (Cat.#: DF2375, 1:1,000, Proteintech, China). After incubation, blots were rinsed five times for 15 min each with TBST, and excess liquid was removed. Finally, the membranes were sealed in plastic wrap and processed using suitable detection methods.

## Results

### Characterization of a photoaging model in SD rats

**FIGURE 1:**
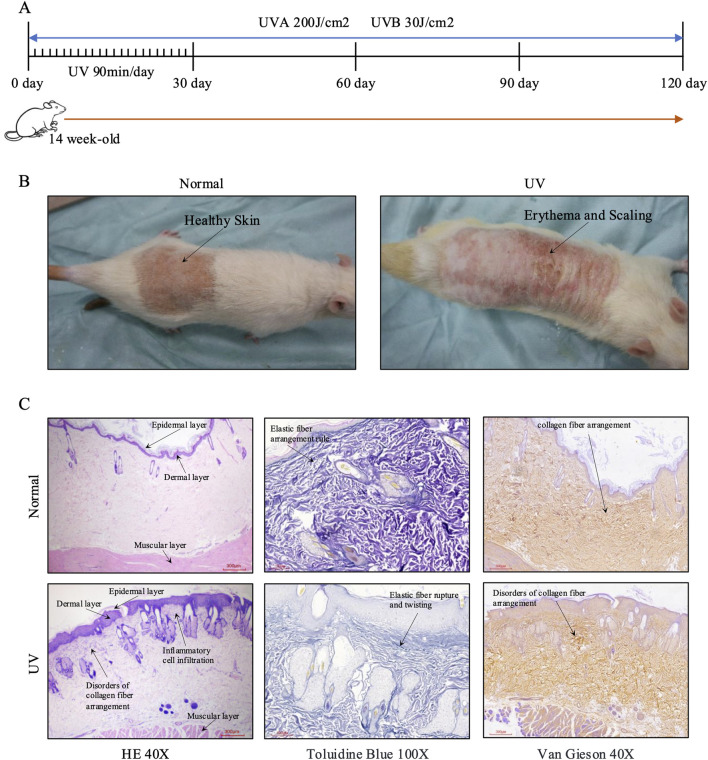
Development of the rat skin photoaging model and histological changes in photoaged rat skin. **(A)** Timeline illustrating the progression of the skin photoaging model. **(B)** General observations of rat skin in control and UV-exposed groups, highlighting photoaging effects. The control group (Normal) shows healthy skin with an intact surface and uniform pigmentation, while the UV-exposed group exhibits pronounced erythema, scaling, and rough texture, indicative of photoaging. **(C)** Histological changes in control and UV-damaged rat skin on day 120, as observed with hematoxylin and eosin (HE), toluidine blue, and Van Gieson staining. In the control group (Normal), HE staining (×40) reveals a well-organized epidermal layer, dermal layer, and muscular layer with regular elastic fiber arrangement (toluidine blue, ×100) and normal collagen fiber arrangement (Van Gieson, ×40). In the UV-exposed group, HE staining (×40) shows epidermal hyperplasia, inflammatory cell infiltration, disorders of collagen fiber arrangement, and an intact muscular layer. Toluidine blue staining (×100) highlights elastic fiber rupture and twisting, while Van Gieson staining (×40) confirms disordered collagen fiber arrangement. Scale bars are provided in each image: ×40 images indicate 300 μm, while ×100 images indicate 100 μm. Abbreviations: HE, Hematoxylin and Eosin; UV, Ultraviolet.

A total of 20 SD rats were split into two groups: the UV-group (n = 10) and the negative control group (n = 10). After 120 days of exposure to UVA and UVB radiation (Xu et al., 2022), the UV-group displayed marked signs of photoaging (Figure 1A). Clinical assessments of the UV-exposed rats showed significant skin changes such as increased dryness, laxity, fine lines, surface roughness, and enlarged pores (Figure 1B). This group also showed pigmentation irregularities, including both hyperpigmentation and hypopigmentation, as well as vascular changes like dilated epidermal blood vessels. Histopathological evaluation using hematoxylin and eosin (HE) staining confirmed the morphological alterations linked to photoaging (Figure 1C). In the UV-group, the epidermal layer showed uneven thickening with scattered atrophic areas. The dermis was characterized by disordered and expanded vascular networks, a substantial decrease in type I collagen, and an upsurge in reticular fibers. Moreover, there was a noticeable degeneration and aggregation of elastic fibers, along with significant cellular infiltration in the photoaged dermis. In contrast, the control group exhibited a well-maintained epidermal structure featuring uniformly distributed sebaceous glands (Figure 1C). Specialized staining of elastic fibers in the UV-treated skin showed considerable fragmentation and a reduction in fiber density, unlike the well-ordered structure seen in the control group (Figure 1C). Additionally, staining of collagen fibers in the UV-treated skin displayed localized proliferation and clumping, with signs of fragmentation and disorganization. Conversely, collagen fibers in the control group skin were consistently arranged without evidence of damage or disarray.

These observations collectively validate the establishment of a photoaging model in SD rats, showing clear morphological and histological signs of skin aging.

### Exosome isolation and characterization in photoaging

**FIGURE 2:**
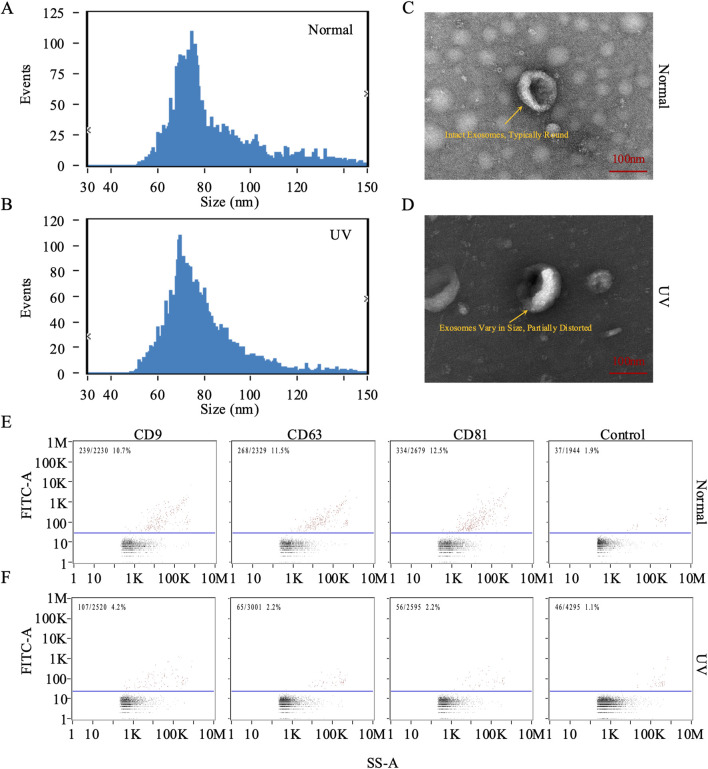
Characterization of exosomes from rat skin. **(A, B)** Quantification and size distribution of exosomes analyzed by nanoparticle tracking analysis (NTA). **(C, D)** Representative transmission electron microscopy (TEM) images of exosomes isolated from skin tissue. In the control group (Normal), panel **(C)** shows intact exosomes with a typically round morphology, displaying a characteristic ring-like structure with a diameter of approximately 100 nm (scale bar: 100 nm). In the UV-exposed group, panel **(D)** reveals exosomes that vary in size and are partially distorted, exhibiting irregular shapes and heterogeneous internal density, indicative of UV-induced morphological changes (scale bar: 100 nm). **(E, F)** Representative plots of CD9, CD63, and CD81 on individual exosomes were assessed using FITC and PE-conjugated antibodies via nano flow cytometry (NanoFCM analysis). Bivariate dot plots depict fluorescence intensity *versus* side scatter (SS-A).

Exosomes are crucial in the skin photoaging process. To explore the effects of UV exposure on exosome characteristics, we isolated exosomes from the dorsal skin of both the control and UV-exposed rat groups and analyzed their size and morphology. Using NTA, we identified a distinct peak at approximately 70 nm for both groups. The average diameters were recorded as 83.86 nm for the control group and 78.8 nm for the UV-exposed group (Figures 2A, B), indicating a slight increase in size for the control group. TEM further confirmed their characteristic size and morphology (Figures 2C, D). Additionally, nanoscale flow cytometry was employed to measure the expression of exosomal markers CD63, CD9, and CD81, which indicated increased positivity rates for these markers in both groups compared to baseline levels (Figures 2E,F). These detailed analyses confirm the successful isolation and characterization of exosomes from rat skin tissue.

### Transcriptomic profiling of exosomes from UV-Exposed skin tissue

**FIGURE 3:**
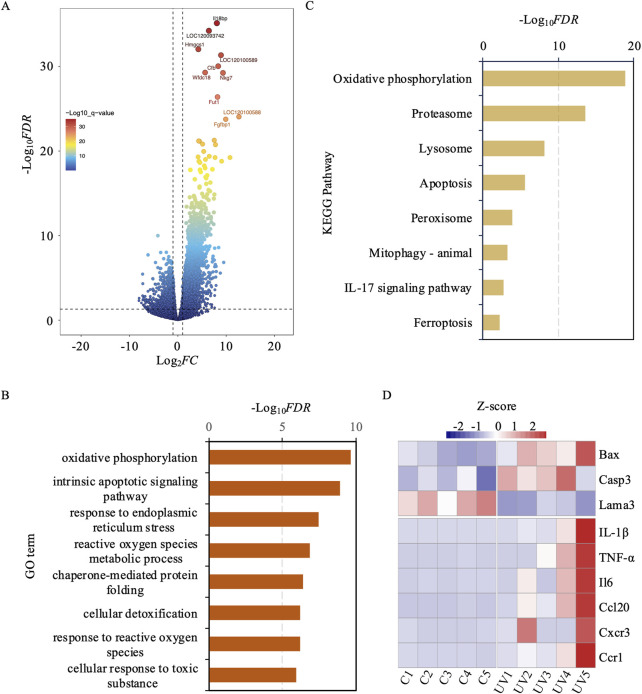
Differential gene expression, functional enrichment, and kegg pathway analysis between uv-induced and control groups. **(A)** Volcano plot illustrating differentially expressed genes between the UV-induced and control groups. Red dots indicate high significance, while blue dots indicate low significance. **(B)** GO enrichment analysis of differentially expressed genes. The y-axis represents GO terms, and the x-axis represents -log_10_ *FDR*. **(C)** KEGG Pathway enrichment analysis of differentially expressed genes. The x-axis represents -log_10_ *FDR*, and the y-axis denotes KEGG pathway terms. **(D)** The heatmap reveals that several genes, particularly IL-1β, TNF-α, and IL6, exhibit marked upregulation under UV exposure conditions.

To understand the transcriptomic landscape of exosomes derived from UV-exposed murine skin, we performed mRNA sequencing analysis. This analysis revealed significant changes in the transcriptomic profile, with 5,906 mRNAs differentially expressed, including 1,667 downregulated and 4,239 upregulated genes (Figure 3A; Supplementary Table S1).

To decipher the biological implications of these transcriptomic shifts, we performed a gene ontology (GO) analysis on the DEmRNAs (Figure 3B). This analysis revealed a significant enrichment of pathways linked to the exosomal response to UV-induced stress, shedding light on the molecular mechanisms involved in skin photoaging. Key pathways enriched included oxidative phosphorylation, intrinsic apoptotic signaling, response to endoplasmic reticulum stress, and ROS metabolic processes. These pathways play critical roles in managing cellular responses to oxidative stress and damage, which are crucial in the development of skin photoaging. Additionally, pathways involved in chaperone-mediated protein folding, cellular detoxification, and responses to ROS and toxic substances were also highlighted. These findings suggest an adaptive boost in cellular defenses, aimed at maintaining cellular integrity and functionality in the face of UV-induced environmental stress.

Simultaneously, we performed a KEGG pathway enrichment analysis to delve deeper into the molecular mechanisms driving skin photoaging (Figure 3C). This analysis underscored the critical role of pathways such as oxidative phosphorylation, which is essential for ATP production and may be compromised by oxidative stress. It also showed increased activity in the proteasome pathway, suggesting an elevated need for protein degradation and turnover due to UV-induced protein damage. The lysosomal pathway was also prominent, playing a crucial role in the cellular breakdown processes to eliminate damaged organelles and proteins. Additionally, apoptosis-related pathways were evident, indicating the potential for UV-induced cell death in aging skin. The analysis also highlighted the significance of peroxisomes in lipid metabolism and ROS detoxification to combat oxidative stress. Furthermore, the process of mitophagy was emphasized as crucial for selectively degrading damaged mitochondria to preserve cellular health. The IL-17 signaling pathway was linked to inflammatory responses that might intensify skin damage, while ferroptosis emerged as a critical, regulated form of cell death associated with oxidativestress. Overall, these KEGG pathways depict a sophisticated network of cellular responses to UV-induced damage, highlighting the complex and adaptive strategies skin cells employ to mitigate environmental stressors.

In addition to these global transcriptomic shifts, a targeted analysis revealed nine key genes implicated in apoptosis and inflammation (Figure 3D). Specifically, Bax and Casp3 were significantly upregulated, while Lama3 was downregulated, collectively suggesting a pro-apoptotic trend in response to UV-induced stress. Concurrently, six inflammation-related genes (Il1b, Tnf, Il6, Ccl20, Cxcr3, and Ccr1) were markedly upregulated, indicating an enhanced inflammatory response. These targeted findings support and extend the broader GO and KEGG pathway analyses by underscoring the pivotal roles of apoptosis and inflammation in the exosomal cargo, thus highlighting exosomes as mediators of photoaging through their potential to modulate both cell death and inflammatory cascades.

### Cis-regulatory target gene analysis of lncRNAs following UV exposure

**FIGURE 4:**
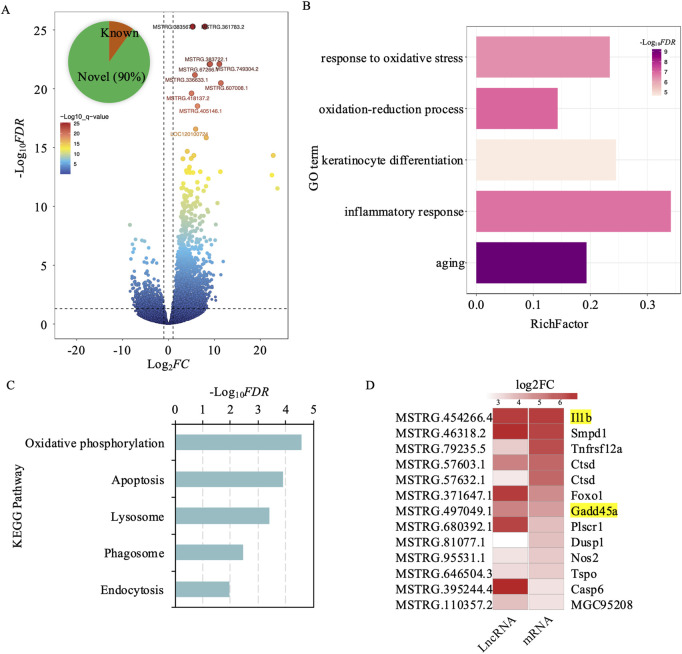
Differential expression analysis of cis-regulated target genes by lncrnas in exosomes from UV-induced and control groups. **(A)** Volcano plot depicting differentially expressed lncRNAs between the UV-induced and control groups. Red dots indicate high significance, and blue dots indicate low significance. **(B)** GO enrichment analysis of 2,336 differentially expressed genes cis-regulated by lncRNAs. The x-axis represents the enrichment factor, defined as the ratio of differentially expressed genes to all genes annotated to the GO terms. Purple indicates higher significance. The y-axis represents GO terms. **(C)** KEGG enrichment analysis of 2,336 differentially expressed genes cis-regulated by lncRNAs. The x-axis represents -log_10_ *FDR*, and the y-axis denotes KEGG pathway terms. **(D)** Trends in lncRNA and gene expression changes involved in apoptosis following UV-induction.

To explore the changes in lncRNA profiles within exosomes due to UV exposure, we carried out a comprehensive lncRNA sequencing study. This analysis detected 2,332 lncRNAs that showed differential expression (DElncRNAs), with 539 being downregulated and 1,793 upregulated. Notably, about 90% of these lncRNAs, amounting to 2,100, were previously uncharacterized, suggesting a significant expansion of the lncRNA repertoire following UV radiation exposure (Figure 4A; Supplementary Table S2). To understand the influence of DElncRNAs on mRNA expression and their biological functions, we examined their regulatory interactions with adjacent protein-coding genes located within a 100 kb radius. Our cis lncRNA-mRNA interaction analysis revealed 9,087 gene pairs involving 2,336 differentially expressed genes, with 1,913 showing upregulation (Supplementary Table S3).

GO enrichment analysis of these adjacent genes showed significant links to pathways associated with aging, oxidative stress response, inflammation, keratinocyte differentiation, and oxidation-reduction processes. These results emphasize the complex regulatory networks orchestrated by DElncRNAs, underscoring their crucial role in shaping cellular responses to UV-induced skin damage and aging. Further insights into the molecular mechanisms of skin photoaging were provided by KEGG pathway enrichment analysis. Key pathways identified include oxidative phosphorylation, which may be compromised by UV-induced oxidative stress, impacting ATP production (Figure 4B). The enrichment of the proteasome pathway suggests an increase in protein degradation and turnover as a result of UV damage, whereas the lysosomal pathway is vital for cellular degradation processes, assisting in the removal of damaged organelles and proteins. Apoptosis-related pathways indicate a likely increase in cell death triggered by UV exposure. Furthermore, endocytosis was emphasized for its importance in nutrient absorption and the regulation of cellular signaling, which are crucial for maintaining cellular homeostasis (Figure 4C).

Among the 9,087 cis lncRNA-mRNA interaction pairs identified, 5,197 involved both DElncRNAs and DEmRNAs. To further validate these regulatory interactions, we created a correlation matrix based on gene expression levels and found 1,327 significant interaction pairs (Pearson correlation *r* ≥ ∣0.80|; *p* < 0.05; Supplementary Table S4). Of these, 97% (1,293 pairs) exhibited positive correlations, indicating that the expression of lncRNAs and their corresponding mRNAs tended to shift in the same direction—either both upregulated or both downregulated. Notably, in 1,280 of these pairs, both the lncRNA and mRNA were concurrently upregulated, suggesting that these lncRNAs may act to enhance the expression of their target mRNAs. The 1,280 gene pairs identified in this study comprised 1,060 unique genes, which underwent functional enrichment analysis to elucidate their biological roles. The analysis revealed that these lncRNA-associated genes are involved in several critical cellular processes, including the regulation of organelle function, enhancement of protein binding to support signal transduction and structural integrity, activation of inflammatory and immune responses, and positive regulation of the apoptotic process (Supplementary Table S5). These findings underscore the pivotal role of lncRNAs in modulating these pathways, suggesting that cells respond to UV-induced damage through lncRNA-mediated regulation of organelle function, reinforcement of protein interactions, initiation of inflammatory and immune defenses, and promotion of apoptosis to facilitate repair and adaptation mechanisms. Specifically, within these pathways, genes associated with apoptosis, such as IL-1B and GADD45B, exhibited consistent upregulation and strong positive correlations with their interacting lncRNAs (Figure 4D). Here, IL-1B serves as a key mediator of inflammatory responses and cell proliferation, while GADD45B contributes to the regulation of cell growth and apoptosis. These observations reinforce the critical role of exosomal DElncRNAs in orchestrating intricate regulatory networks that modulate gene expression, thereby shaping cellular responses to UV-induced stress and damage.

### Investigating the role of *IL-1B* and *GADD45B* in UV-Induced cellular response

**FIGURE 5:**
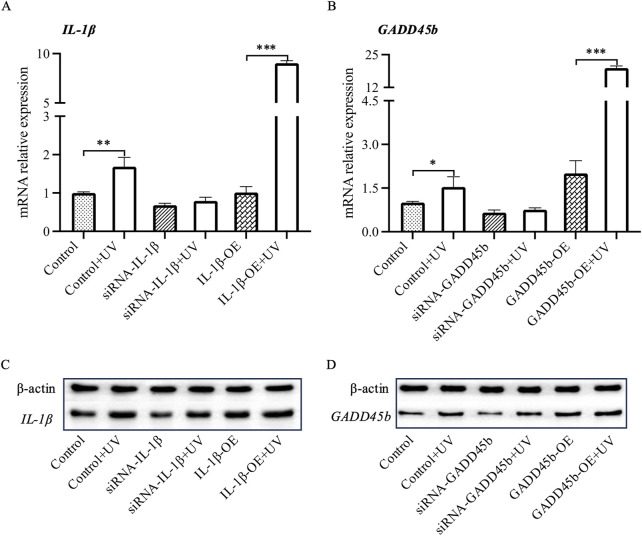
RT-PCR and Western blot analysis of Il1b and Gadd45a mRNA and protein levels in HaCaT cells. Following siRNA-mediated silencing and overexpression of Il1b and Gadd45a in HaCaT cells, mRNA and protein expression levels of both genes were assessed UV-induction using RT-PCR and Western blot analysis. **(A, B)** mRNA expression analysis. **(C, D)** Protein expression analysis. * *P*-value < 0.05, ** *P*-value < 0.01, *** *P*-value < 0.001.

In our previous studies, we had observed significant changes in *IL-1B* and *GADD45B*, which are crucial targets influenced by lncRNAs after UV exposure. To further investigate the cellular functions of these genes under UV conditions, we carried out experiments using human immortalized keratinocytes (HaCaT cells). RT-qPCR analysis showed a significant increase in the expression of *IL-1B* and *GADD45B* post-UV exposure (Figures 5A, B). Further experiments involving overexpression and inhibition assays for these genes were conducted. Upon UV irradiation, overexpression led to a significant elevation in their expression levels (Figures 5A, B), while inhibition resulted in negligible changes, indicating a variable response mechanism. These results were supported by Western blot analysis, which demonstrated substantial increases in protein levels with overexpression, but minimal changes with inhibition (Figures 5C, D). Together, these findings deepen our understanding of roles of *IL-1B* and *GADD45B* in cellular responses to UV stress, highlighting their potential involvement in UV-induced signaling pathways.

**FIGURE 6:**
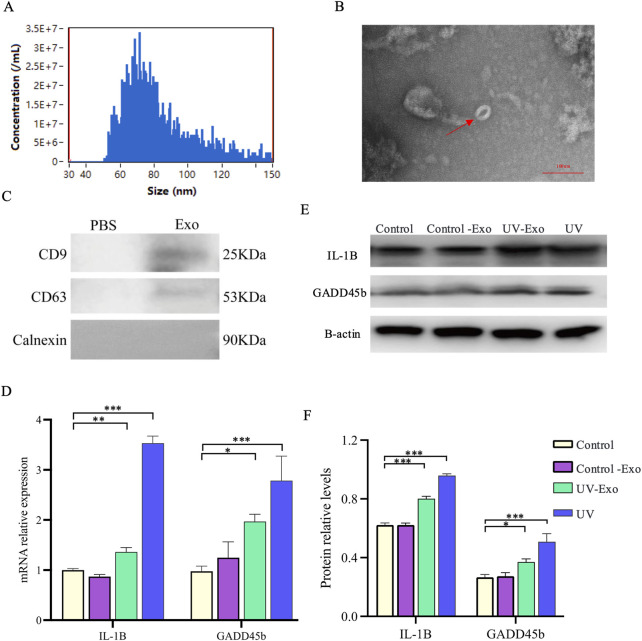
Exosome characterization and transmission of UV-induced IL-1β and GADD45B expression Changes in HaCaT Cells. **(A)** Particle size distribution of exosomes isolated from HaCaT cells, determined by nanoparticle tracking analysis. **(B)** Representative TEM image of exosomes, revealing characteristic cup-shaped vesicles (indicated by a red arrow). Scale bar = 100 nm. **(C)** Exosome-enriched protein markers. Including CD9 (25 kDa) and CD63 (53 kDa). And a neaative marker. Calnexin (90 kD) were analyzed by Western blot. **(D)** Relative mRNA expression levels of IL-1β and GADD45B were assessed by RT-qPCR in recipient HaCaT cells from four experimental groups, namely, untreated control, Control-Exo (treated with exosomes from untreated cells), UV-Exo (treated with exosomes from UV-irradiated cells), and direct UV exposure. **(E)** Western blot analysis of IL-1β and GADD45B protein levels in the same experimental groups, with β-actin as a loading control. **(F)** Quantification of relative IL-1β and GADD45B protein levels, normalized to the control group. * *P*-value < 0.05, ** *P*-value < 0.01, *** *P*-value < 0.001.

To investigate whether UV-induced changes in IL-1β and GADD45B expression can be transmitted via exosomes, we evaluated the effects of exosomes derived from UV-exposed HaCaT keratinocytes on recipient HaCaT cells, using direct UV exposure as a comparator. Exosomes were isolated from HaCaT cells that were either subjected to UV irradiation (UV-Exo) or left untreated (Control-Exo). The identity and purity of the isolated exosomes were rigorously validated through multiple characterization techniques. Particle size analysis demonstrated a distribution peaking between 50 and 100 nm, consistent with the typical exosome size range of 30–150 nm (Figure 6A). TEM further confirmed the presence of intact, cup-shaped vesicles with diameters of approximately 60 nm (Figure 6B). Additionally, Western blot revealed enrichment of exosome-specific markers CD9 (25 kDa) and CD63 (53 kDa), and confirmed the absence of detectable Calnexin (90 kDa) (Figure 6C). These comprehensive characterizations establish the reliability of the exosome preparations used in subsequent experiments.

Recipient HaCaT cells were assigned to four experimental groups: untreated control, Control-Exo-treated, UV-Exo-treated, and direct UV exposure. Quantitative RT-qPCR analysis revealed a significant upregulation of IL-1β and GADD45B mRNA levels in both the UV-Exo group (P < 0.01) and the direct UV exposure group (P < 0.001) compared to the untreated control (Figure 6D). In contrast, the Control-Exo group showed mRNA expression levels comparable to the untreated control, indicating that exosomes from untreated cells do not induce these changes. These transcriptional findings were corroborated at the protein level by Western blot analysis, which exhibited markedly elevated IL-1β and GADD45B protein expression in the UV-Exo and direct UV exposure groups, whereas the Control-Exo group showed expression levels comparable to the Control group and lower than those in the UV-Exo and direct UV exposure groups (Figure 6E). Equal protein loading across all samples was confirmed by consistent β-actin levels. Quantitative analysis of the Western blot data further validated these observations, showing significant increases in the relative protein levels of IL-1β and GADD45B in the UV-Exo and UV groups compared to the Control and Control-Exo groups (Figure 6F).

These results align with our previous observations (Figure 5), which indicated a significant increase in IL-1β and GADD45B expression following direct UV exposure. Collectively, these data suggest that UV-induced upregulation of IL-1β and GADD45B can be mediated by exosomes, implying that UV-Exo may contain regulatory molecules capable of inducing a UV-like stress response in recipient cells or enhancing the broader tissue response to UV exposure.

### UV-exposed exosomes trigger apoptosis and inflammation in HaCaT cells

Based on our prior observation that exosomes derived from UV-Exo significantly upregulate the inflammation-related genes IL-1β and GADD45B in recipient HaCaT keratinocytes, we sought to determine whether UV-Exo could elicit broader molecular changes comparable to those induced by direct UV exposure. To address this, we evaluated HaCaT cells across four experimental groups, namely, Control, Control-Exo, UV-Exo, and UV. The effects on apoptosis and inflammation were assessed using RT-PCR, Western blot, and ELISA techniques. For apoptosis, we examined the key markers Bcl-2, Caspase 3, and p53, while for inflammation, we focused on IFN-γ, TNF-α, and IL-6.

**FIGURE 7:**
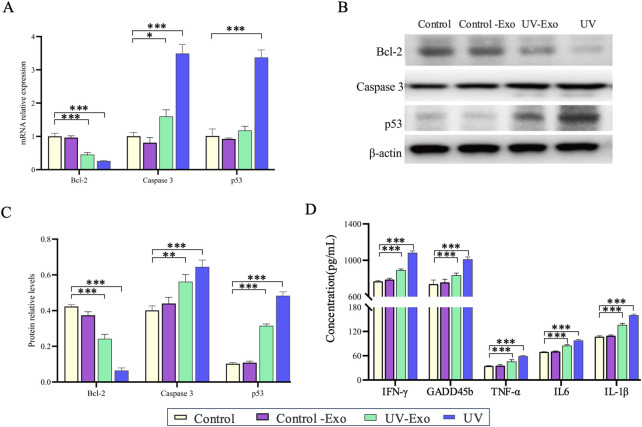
Effects of UV-Exo and direct UV exposure on apoptosis and inflammation in HaCaT cells. **(A)** Relative mRNA expression levels of Bcl-2, Caspase 3, and p53 in HaCaT cells across four groups: Control, Control-Exo, UV-Exo, and UV. Data were assessed by RT-PCR. **(B)** Western blot analysis of Bcl-2, Caspase 3, and p53 protein levels in HaCaT cells from the four experimental groups: Control, Control-Exo, UV-Exo, and UV. β-actin serves as the loading control. **(C)** Relative protein levels of Bcl-2, Caspase 3, and p53 in HaCaT cells across the four groups, quantified by densitometry and normalized to β-actin. **(D)** Concentrations of inflammatory cytokines (IFN-γ, GADFβ, TNF-α, IL-6, IL-1β) in culture supernatants of HaCaT cells from the four groups, measured by ELISA. * *P*-value < 0.05, ** *P*-value < 0.01, *** *P*-value < 0.001.

RT-PCR analysis revealed that UV-Exo treatment significantly downregulated the anti-apoptotic gene Bcl-2 while markedly upregulating the pro-apoptotic genes Caspase 3 and p53, closely mirroring the expression patterns observed in the UV group. In contrast, the Control-Exo group showed minimal changes in these genes (Figure 7A). Western blot analysis further confirmed these findings, demonstrating a significant reduction in Bcl-2 protein levels and pronounced increases in Caspase 3 and p53 protein levels in both the UV-Exo and UV groups, whereas no notable differences were observed between the Control-Exo and Control groups (Figures 7B, C). Additionally, ELISA assays indicated that secretion of inflammatory cytokines (IFN-γ, TNF-α, and IL-6) was significantly elevated in the UV-Exo and UV groups, while levels in the Control-Exo group remained comparable to those in the Control group (Figure 7D).

In summary, UV-Exo induces cellular responses akin to those triggered by direct UV exposure by modulating the mRNA expression and protein levels of apoptosis- and inflammation-related genes. These findings suggest that UV-Exo plays a pivotal role in propagating UV-induced cellular stress responses.

## Discussion

This study underscores the pivotal role of lncRNAs in mediating cellular responses to UV-induced stress through their presence in exosomes, revealing a complex network of interactions between differentially expressed DElncRNAs and adjacent protein-coding genes. This network highlights the significant role of lncRNAs in adjusting cellular reactions to UV exposure. Previous studies have documented the aberrant expression of lncRNAs in skin cells under UV stress, affecting multiple target genes and engaging in various pathways such as aging, melanogenesis, collagen synthesis, and the MAPK and TGF-β signaling pathways (D'Mello et al., 2016; Zheng et al., 2017; Piipponen et al., 2020; Ke and Wang, 2021). These studies have established lncRNAs as key regulators of skin photoaging. For instance, lncRNAs can modulate the expression of matrix metalloproteinases (MMPs), which are crucial for collagen degradation, thereby influencing skin elasticity and integrity (Guo et al., 2022; Zhao et al., 2022). Moreover, lncRNAs play a role in managing oxidative stress pathways by interacting with antioxidant response elements, enhancing cellular defense mechanisms against UV damage (Yang et al., 2023). In our investigation, we identified that lncRNAs within exosomes are vital for orchestrating cellular stress responses to UV radiation, with exosomes acting as carriers that transfer lncRNAs to recipient cells, thereby amplifying the cellular response to stress (Elsharkasy et al., 2020). The interaction networks between DElncRNAs and protein-coding genes are significantly enriched in GO terms associated with aging, oxidative stress response, inflammation, and cell differentiation, highlighting their alignment with the known impacts of UV radiation on skin cells and emphasizing the role of lncRNAs in aiding cells adapt and mitigate UV-induced damage (Lin et al., 2021).

Our correlation analysis uncovered 1,327 significant interaction pairs between lncRNAs and mRNAs, underscoring the substantial regulatory impact of lncRNAs. Interestingly, 97% of these pairs (1,293) exhibited positive correlations, suggesting that lncRNAs may predominantly enhance the expression of their target mRNAs, amplifying their regulatory influence in cellular processes. Notably, genes linked to apoptosis such as *IL-1B* and *GADD45B* were consistently found to be upregulated in conjunction with their corresponding lncRNAs, suggesting a synchronized regulatory mechanism that facilitates apoptosis in response to UV damage. This synchronized upregulation further supports the idea that lncRNAs may enhance the expression of their target mRNAs, such as IL-1B and GADD45B, thereby promoting the cellular stress response tied to apoptosis and inflammation. This observation aligns with prior research that illustrates lncRNAs’ role in governing apoptosis and inflammatory responses (Jiang et al., 2021; Zhang et al., 2024). For instance, certain lncRNAs are known to influence the transcriptional regulation of factors like Sirt1, thereby modulating the expression of pro-inflammatory cytokines and affecting inflammatory processes (Yang et al., 2020). We further corroborated these results in HaCaT cells, observing that UV exposure prompted an increase in the expression levels of *IL-1B* and *GADD45B*, which confirms their involvement in the cellular response to stress. These findings suggest that targeting lncRNA interactions may serve as a viable approach to reduce UV-induced skin damage.

In our study on lncRNA cis-regulation, we identified the previously reported long non-coding RNA (lncRNA) TINCR, which is upregulated in a TP53-dependent manner following ultraviolet (UV) exposure in human keratinocytes (Morgado-Palacin et al., 2023). Our analysis revealed that TINCR and the adjacent gene MICOS13 exhibited synchronized upregulation after UV exposure (r = 0.8, p < 0.005), indicating a cis-regulatory role in enhancing inflammation-related gene expression. Literature further shows that Tincr-deficient mice display impaired resolution of inflammatory responses following UVB irradiation, with increased neutrophilic microabscesses. These findings align with our observations, enhancing the credibility of our research on lncRNA-mediated cis-regulation in UV-induced cellular stress responses.

Exosomal lncRNAs play a vital role in intercellular communication and modulating stress responses (Valadi et al., 2007; Pegtel et al., 2010; Men et al., 2019). Previous studies have explored the function of exosomal lncRNAs in various stress conditions, such as oxidative stress and inflammation (Li et al., 2024). Our study builds on this foundation by specifically connecting exosomal lncRNAs to cellular adaptations induced by UV exposure, underscoring their potential as therapeutic targets for addressing skin aging and damage. Furthermore, the cargo of exosomes derived from skin cells exhibits distinct characteristics that distinguish them from those of other tissues. It is well-established that exosomes from various cell types encapsulate cargo, including proteins and nucleic acids, which mirror their cellular origins and physiological functions. For instance, skin-derived exosomes, particularly those secreted by keratinocytes, are enriched with biomolecules that promote angiogenesis and fibroblast activity, thereby facilitating wound healing (Xiong et al., 2021). In contrast, proteomic analyses reveal significant differences in protein composition between exosomes from blood and urinary sources. Specifically, urine-derived exosomes are enriched with proteins such as podocin and AQP2, indicative of their renal origins from glomerular podocytes and collecting duct cells (Schey et al., 2015). These proteins, however, are either undetectable or present at negligible levels in blood-derived exosomes, which instead contain abundant serum proteins like albumin. This tissue-specific cargo composition suggests that skin-derived exosomes contain molecules adapted to skin physiology, emphasizing their potential as specialized therapeutic agents or biomarkers for combating photoaging.

In conclusion, the complex regulatory networks orchestrated by DElncRNAs within exosomes highlight their crucial role in cellular responses to UV-induced stress. These findings enhance our understanding of the molecular mechanisms involved in skin photoaging and offers valuable insights for devising lncRNA-based therapeutic approaches aimed at combating UV-related skin damage. Overall, By examining how DElncRNAs facilitate communication and response adjustments among cells under stress, our observations pave the way for potential interventions that could mitigate the effects of UV exposure, thus preserving skin health and appearance.

## Data Availability

The datasets presented in this study can be found in online repositories. The names of the repository/repositories and accession number(s) can be found in the article/Supplementary Material.

## Abbreviations

DElncRNAs, differentially expressed lncRNAs; DEmRNAs, differentially expressed mRNAs; HE, hematoxylin and eosin; LncRNA, long non-coding RNAs; mRNA, messenger RNAs; NCBI, national center for biotechnology information; ROS, reactive oxygen species; SD, Sprague Dawley; TPM, transcripts per million; UV, ultraviolet.
